# fMRI orientation decoding in V1 does not require global maps or globally coherent orientation stimuli

**DOI:** 10.3389/fpsyg.2013.00493

**Published:** 2013-08-12

**Authors:** Arjen Alink, Alexandra Krugliak, Alexander Walther, Nikolaus Kriegeskorte

**Affiliations:** Medical Research Council, Cognition and Brain Sciences UnitCambridge, UK

**Keywords:** pattern analysis, orientation selectivity, fMRI, visual cortex, global form, hyperacuity, decoding, radial bias

## Abstract

The orientation of a large grating can be decoded from V1 functional magnetic resonance imaging (fMRI) data, even at low resolution (3-mm isotropic voxels). This finding has suggested that columnar-level neuronal information might be accessible to fMRI at 3T. However, orientation decodability might alternatively arise from global orientation-preference maps. Such global maps across V1 could result from bottom-up processing, if the preferences of V1 neurons were biased toward particular orientations (e.g., radial from fixation, or cardinal, i.e., vertical or horizontal). Global maps could also arise from local recurrent or top-down processing, reflecting pre-attentive perceptual grouping, attention spreading, or predictive coding of global form. Here we investigate whether fMRI orientation decoding with 2-mm voxels requires (a) globally coherent orientation stimuli and/or (b) global-scale patterns of V1 activity. We used opposite-orientation gratings (balanced about the cardinal orientations) and spirals (balanced about the radial orientation), along with novel patch-swapped variants of these stimuli. The two stimuli of a patch-swapped pair have opposite orientations everywhere (like their globally coherent parent stimuli). However, the two stimuli appear globally similar, a patchwork of opposite orientations. We find that all stimulus pairs are robustly decodable, demonstrating that fMRI orientation decoding does not require globally coherent orientation stimuli. Furthermore, decoding remained robust after spatial high-pass filtering for all stimuli, showing that fine-grained components of the fMRI patterns reflect visual orientations. Consistent with previous studies, we found evidence for global radial and vertical preference maps in V1. However, these were weak or absent for patch-swapped stimuli, suggesting that global preference maps depend on globally coherent orientations and might arise through recurrent or top-down processes related to the perception of global form.

## Introduction

Visual orientation information is thought to be represented in fine-scale columnar preference patterns in early visual cortex. Despite the sub-millimeter grain of V1 orientation columns, it has been shown that fMRI, at standard resolution (3 mm isotropic), enables us to decode the orientation of a uniform visual grating from V1 (Kamitani and Tong, 2005). Orientation sensitivity of 3-mm fMRI voxels could result from subtle biases in each voxel's sample of columnar selectivities (Haynes and Rees, 2005; Kamitani and Tong, 2005). This idea had a big impact because it suggests that standard-resolution fMRI in humans allows us to decode columnar-scale neuronal representations.

But do fMRI patterns really reflect columnar-scale neuronal representations? Alternatively, fMRI orientation decoding could rely entirely on coarse-scale neuronal organizations, with no contribution from the columnar scale at all (Op de Beeck, 2010a). This issue has sparked significant debate (Gardner, 2010; Kamitani and Sawahata, 2010; Kriegeskorte et al., 2010; Op de Beeck, 2010b; Shmuel et al., 2010; Swisher et al., 2010). A particular coarse-scale organization that might account for V1 orientation decoding is a global radial-preference map (Sasaki et al., 2006). If V1 has a radial-preference map, a grating will elicit stronger feed-forward activation in V1 patches representing visual field regions where the grating's edges point toward fixation. Both evidence for (Freeman et al., 2011) and against (Mannion et al., 2009; Seymour et al., 2010) this account has been provided by recent neuroimaging studies.

The discussion of these issues in the literature has tacitly assumed that it is feed-forward processing of visual orientation that gives rise to the decoded signals (whether they reflect fine-grained or global orientation-preference maps). However, the cited studies used uniform gratings, where orientations are globally coherent across space and different orientations give rise to distinct global-form percepts. For example, a left-tilted and a right-tilted grating are associated with very different global-form percepts. The possibility that global-form-related effects, including pre-attentive grouping, attention spreading, and global-form representation, contribute to fMRI orientation decodability has not been addressed. Recurrent processing, through lateral connectivity within V1 or through feedback from higher regions representing the stimuli more holistically (Pasupathy and Connor, 2002; Kourtzi and Huberle, 2005; Ostwald et al., 2008), could influence the V1 representation.

**Figure 1:**
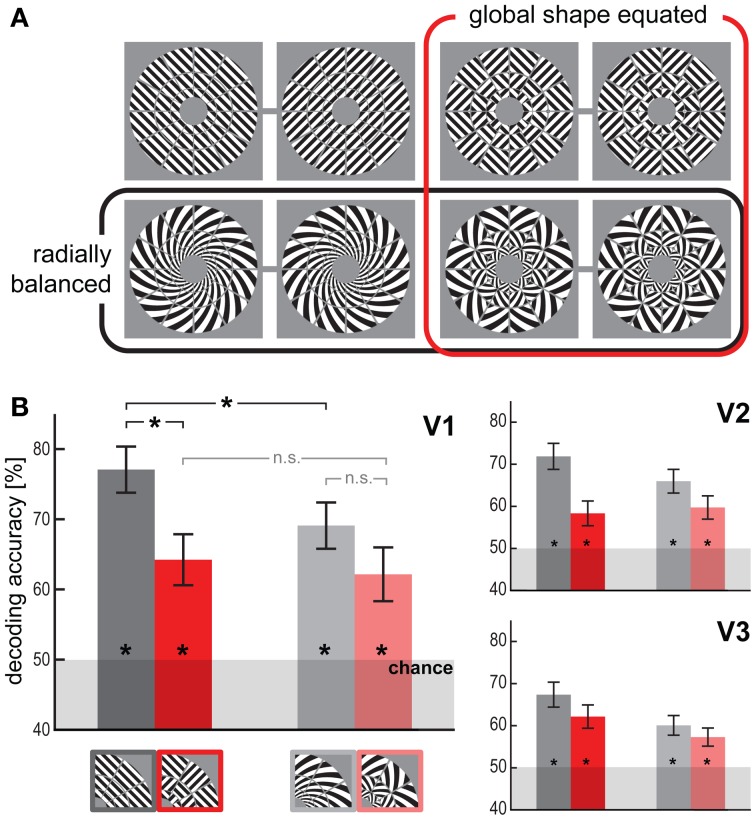
**Visual orientations are robustly decodable for all stimulus types. (A)** The four stimulus types, uniform gratings (upper left pair), spirals (lower left pair), patch-swapped gratings (upper right pair), and patch-swapped spirals (lower right pair). For each type we presented two differently oriented exemplars (pairing indicated by gray lines) with a 90-deg orientation disparity at every location. Stimuli were presented centered on fixation. The retinal diameter of each stimulus was 14.08° (inner-border radius: 1.5°, outer-border radius: 7.04°). **(B)** Orientation decoding accuracy (linear SVM, leave-one-subrun-out cross-validation) for each stimulus type and visual area (V1-3). Error bars indicate the standard error of the mean across all 18 subjects. Asterisks on bars indicate that decoding accuracy was significantly above chance level (*p* < 0.01). Asterisks on horizontal brackets indicate significant differences (*p* < 0.05) between decoding accuracies.

The aim of the current fMRI study is to determine how global-form differences in the stimuli and global preference maps in V1 affect fMRI orientation decoding. In order to address the influence of global-form differences, we use stimuli that either (1) have globally coherent orientations and differ in global form or (2) consist in a patchwork of different orientation, such that two stimuli with opposite orientations in each patch appear globally similar (Figure 1A). In order to address the spatial scale, at which the fMRI orientation information resides for each stimulus type, we apply spatial filtering to the patterns and assess how much orientation information is present in each spatial-frequency band.

We used uniform grating stimuli (45° clockwise or 45° anti-clockwise from the vertical) and logarithmic spirals (with 45° orientation disparity to the radius in clockwise or anti-clockwise direction). The gratings are balanced about the cardinal (i.e., vertical and horizontal) orientations. Thus a global preference map for vertical or horizontal orientations will yield an equal global activation pattern for each grating. The spirals are balanced about the radial orientations. Both spiral stimuli (clockwise and anti-clockwise), when centered on fixation, have an equal absolute orientation disparity to the radius (±45°) everywhere. Thus, a global preference map for radial orientations will yield an equal global activation pattern for each spiral (Mannion et al., 2009; Seymour et al., 2010; Clifford et al., 2011). In order to obliterate the global form of the stimuli, we divided these stimuli into a log-polar array of tiles (3 concentric rings, 12 radial wedges). We then swapped half of the tiles (the “black” tiles of a log-polar “checkerboard”) between the gratings to create patch-swapped variants of the gratings. We performed the same procedure for the spirals (Figure 1A).

## Methods

### Stimuli and design

#### Common features of all stimuli

All stimulus types were presented within an annulus (inner radius = 1.5°, outer radius = 7.04°) centered on fixation on a mid-gray background. The annulus was divided into 36 log-polar tiles defined by 12 radial lines emanating from the center at 30° offsets (including vertical and horizontal directions) and two concentric divisions exponentially spaced between the inner and outer radii (radii including inner and outer: 1.50°, 2.51°, 4.20°, 7.04°). This log-polar tiling was apparent in the form of mid-gray “grout lines” present in all stimuli including the globally coherent ones. For each stimulus type there were two exemplars, which had 90° orientation disparity at every location within the annulus. The oriented edges of all stimuli were hard (rectangular, 100% contrast). The phases of the oriented edges were randomized across presentations of the same exemplar.

#### Gratings

The orientation of the gratings was 45° clockwise and 45° anti-clockwise from the vertical. The gratings had a spatial frequency of 1.25 cycles per visual degree. This spatial frequency drives V1 strongly (Henriksson et al., 2008) and ensures that even the smallest tiles of the log-polar array contains more than a full spatial cycle.

#### Spirals

We used logarithmic spirals whose edges were at a constant angle of ± 45° relative to the radius emanating from fixation. The spiral stimuli had 22 rectangular contrast cycles along the perimeter (i.e., 22 black and 22 white spiral rays). This number of cycles along the perimeter was chosen so as to approximately match the spirals' average spatial frequency across radii to that of the uniform gratings. The two spiral exemplars differed in sense: clockwise or anti-clockwise, lending them 90° orientation disparity at every location. Spiral stimuli are radially balanced because clockwise and anti-clockwise spiral stimuli deviate equally (45°), though in opposite directions, from local radial orientations.

#### Patch-swapped variants

Patch-swapped grating and spiral stimuli result from dividing the ring containing the gratings and spirals into a log-polar checkerboard array of patches and swapping half of the patches (e.g., the black fields of the log-polar checkerboard) between the stimuli. This preserves the 90° orientation disparity at every location and the radial or cardinal balancing (for spirals and gratings, respectively). In contrast to the non-patched swapped stimuli, the two exemplars for the patch-swapped stimuli of each stimulus type elicit very similar global-form percepts.

#### Software and visual presentation

Stimuli were created using Matlab (2009a, The MathWorks, Natwick, MA, USA) and presented in the scanner using Presentation (v1.41). During the experiment, stimuli were projected on a frosted screen at the head end of the scanner bore with a refresh rate of 60 Hz.

#### Experimental design

Stimuli were presented to each subject in a single fMRI session comprising eight scanner runs, each of which lasted 8 min. During each run, we presented both exemplars of one stimulus type (e.g., clockwise and anti-clockwise spirals). Subjects were presented with two runs for each stimulus type. Each run was divided into four equal subruns. Each subrun contained six stimulus blocks (three blocks for each exemplar, with exemplars alternating across blocks, and the leading exemplar alternating across subruns). Each block lasted 14 s and contained phase-randomized versions of a single exemplar. During a stimulus block, 28 phase-randomized versions of the exemplar were presented at a frequency of 2 Hz. The stimulus duration was 250 ms, followed by an interstimulus interval (ISI) of 250 ms, during which only the fixation dot and a tiny task-related ring around it was visible (see *Task*, below). The 28 stimuli had random spatial phases, uniformly distributed between 0 and 2 π. Stimulus blocks were separated by 2-s fixation periods and subruns by 24-s fixation periods.

#### Retinotopic mapping stimuli

In order to define regions of interest (ROIs) for V1-3, we presented dynamic grating stimuli designed to optimally drive early visual cortex. Like the main-experimental stimuli, these stimuli were based on a log-polar array (Figure 1A), but without the grout lines and with 20 patches per ring. Each patch contained rectangular gratings with a spatial period of one-third of the patch's radial width. Grating orientation and phase was assigned randomly to each patch. Over time, the phase of the gratings increased continuously (1 cycle per second) resulting in continuous motion in each patch (in different directions). In addition, the orientation of the grating increased in steps of π/6, once each second, resulting in motion direction changes within patches over time. We used five such stimuli, driving different parts of the retinotopic representations in V1-3: (1) a horizontal double-wedge stimulus, spanning a polar-angle range of ± 15° around the horizontal meridian, (2) a vertical double-wedge stimulus of the same kind, (3) a stimulus that covered the region driven by the main-experimental stimulus (1.50°–7.04° eccentricity), (4) a 0.5°-wide ring peripherally surrounding the main-experimental stimulus annulus (7.04°–7.54° eccentricity), and (5) a 0.5°-wide ring inside the annulus (1.00°–1.50° eccentricity). Stimuli were presented in 6-s blocks. This block length was chosen to balance temporal concentration (which increases design efficiency for long blocks due to hemodynamic buildup) and stimulus adaptation (which reduces design efficiency for long blocks due to reduced neuronal responses). The five dynamic stimuli and 6-s fixation periods were all presented 20 times each in a random sequence over a single run lasting 12 min.

### Subjects and task

#### Subjects

Eighteen healthy volunteers (13 female, age range 20–39) with normal or corrected-to-normal vision took part in this fMRI experiment. Before the experiment, participants were introduced to the experimental procedure and informed consent was given. A separate group of 13 healthy volunteers with normal or corrected-to-normal vision took part in the psychophysical experiment (9 female, age range 21–35).

#### Task—fMRI

During all runs, including retinotopic mapping, *s*ubjects were instructed to continuously fixate a central dot (diameter: 0.06° visual angle). Centered on the fixation dot, there was a small black ring (diameter: 0.20°, line width: 0.03°), which had a tiny gap (0.03°) either on the left or right side. The gap switched sides at random moments in time at an average rate of once per 3 s (with a minimum inter-switch time of 1 s). The task of the subject was to continuously report the side of the gap by keeping the left button pressed with the right index finger whenever the gap was on the left side, and by keeping the right button pressed with the right middle finger whenever the gap was on the right side. The task served to enforce fixation and to draw attention away from the stimuli.

#### Task—psychophysics

Participants were seated in front of a laptop and were instructed to use the mouse to drag-and-drop miniature versions of the eight stimulus types presented during the fMRI experiment into a circular area. They were instructed to arrange stimuli such that the relative distances between images reflect their visual dissimilarity. For a maximum of 15 minutes participants arranged subsets of the eight images. Subsets were selected by an adaptive algorithm that aims to provide optimal evidence for dissimilarity estimates. This multi-arrangement method for acquiring similarity judgments is described in detail in Kriegeskorte and Mur (2012).

### Mri measurements and analysis

#### MRI measurements

Functional and anatomical MRI data were acquired with a 3T Siemens Tim-Trio MRI scanner using a 32-channel head coil. During each main run, we acquired 252 volumes containing 31 slices covering the occipital lobe as well as inferior parietal, inferior frontal, and superior temporal regions for each subject using an EPI sequence (*TR* = 2000 ms, *TE* = 30 ms, flip angle =77°, voxel size: 2.0 mm isotropic, field of view: 205 mm; interleaved acquisition, GRAPPA acceleration factor: 2). The same EPI sequence was employed for retinotopic mapping, during which we acquired 360 volumes. For each participant we also obtained a high-resolution (1 mm isotropic) T1-weighted anatomical image using a Siemens MPRAGE sequence.

#### Data preprocessing

Functional and anatomical MRI data were preprocessed using the Brainvoyager QX software package (Brain Innovation, v2.4). The first two EPI images for each run were discarded (affected by T1 saturation effects). After preprocessing (slice-scan-time correction, 3D head-motion correction, linear-trend removal and temporal high-pass filtering removing frequencies below 2 cycles per run), functional data for all subjects were aligned with the individual high-resolution anatomical image and transformed into Talairach space (Talairach and Tournoux, 1988) as a step toward cortex-based analysis in BrainVoyager. After automatic correction for spatial inhomogeneities of the anatomical image, we created an inflated cortex reconstruction for each subject. All ROIs were defined in each individual subject's cortex reconstruction and projected back into voxel space. Note that we did not use Talairach space or a cortex-based common space for ROI definition and within-ROI patterns were analyzed separately in each subject.

#### Retinotopic mapping

**Figure 2:**
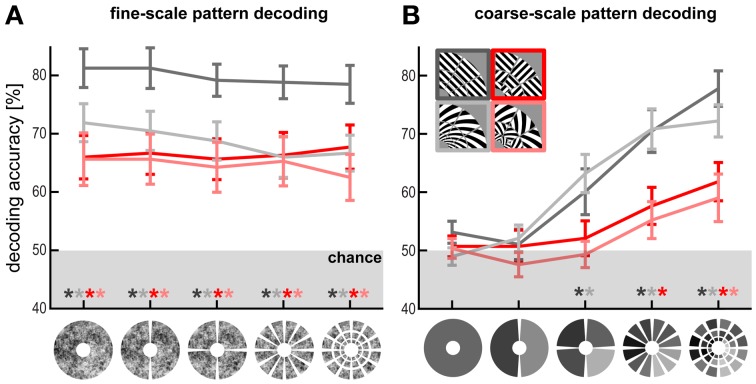
**Fine- and coarse-scale pattern components enable orientation decoding. (A)** Fine-scale pattern decoding. V1 decoding accuracy after subtracting out patch-average activation levels (i.e., removing the spatial low-frequency component) from the patterns at different scales. Patch sizes, from coarse to fine (left to right): full-field representation (1 patch), hemifield representations (2 patches), quarterfield representations (4 patches), 30° radial-wedge representations (12 patches), and each radial wedge divided further into three equally sized cortical patches representing different eccentricities (36 patches). **(B)** Coarse-scale pattern decoding. V1 decoding accuracy based on only the patch averages using the same patch scheme.

A general linear model (GLM) was fitted to the retinotopic mapping data, with five predictors for the five dynamic grating stimuli based on convolving boxcar functions with the hemodynamic response function as described by Boynton et al. (1996). Activation *t*-maps for each stimulus type were projected onto polygon-mesh reconstructions of individual subjects' cortices. We determined the borders between V1-3 based on cortical *t*-maps for responses to vertical and horizontal double-wedge stimuli (Sereno et al., 1995). We defined ROIs for V1-3 as the portion of V1-3 that was more active when presenting the dynamic grating stimulus covering the main-experimental annulus as compared to central and peripheral stimulation (average numbers of voxels for V1-3 ROIs: 1126, 1242, and 1031, respectively, with left and right hemispheres combined). Subsequently, we divided the V1 ROI into 36 equally sized patches, corresponding to a log-polar array in the visual field (Figure 2), which should approximately match the patch division for our main stimuli.

#### Pattern-classifier analyses (Figures 1–3)

Preprocessed functional fMRI data for the main experiment and individual ROI coordinates were imported into Matlab using the NeuroElf Toolbox v0.9c (developed by Jochen Weber, Columbia University). With this toolbox, we computed a GLM for each run of each subject, using one predictor for each exemplar (each of the eight stimuli shown in Figure 1A) for each subrun. We also included six predictors specifying 3D head motion. Each run's GLM, thus, yielded four *t*-value activity patterns for each exemplar (one per subrun). Both runs combined yielded eight *t*-value patterns for each exemplar. We decoded the exemplar (two orientation variants) for each stimulus type with a linear support vector machine (SVM) (Chang and Lin, 2011) using leave-one-subrun-out cross-validation (Mur et al., 2009). We estimated the decoding accuracy separately for each subject. Inference was performed on the set of single-subject accuracies by subtracting the chance-level (50%) and applying the Wilcoxon signed-rank test. To compare accuracies between two stimulus types, we applied the same test to the set of single-subject accuracy differences.

**Figure 3:**
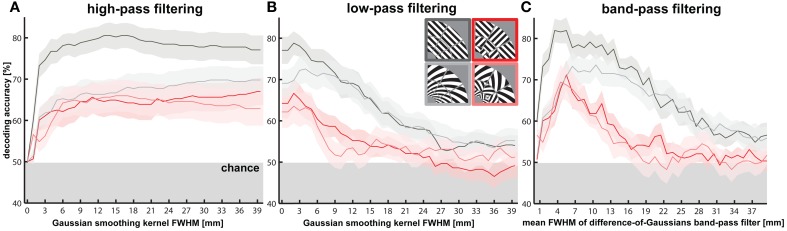
**Effects of spatial high-, low- and band-pass filtering of V1 activity patterns on orientation decodability.** Orientation decodability and SEM (shaded area) is plotted for different levels of spatial **(A)** high- and **(B)** low-pass filtering of V1 activity patterns using a three dimensional Gaussian kernel with a full width at half maximum (FWHM) ranging from 1 to 40 mm in steps of 1 mm. **(C)** Orientation decodability is plotted after spatial band-pass filtering of V1 activity patterns using a bandwidth of 1 mm (difference of FWHMs for difference-of-Gaussians filter) and FWHMs from 1 to 40 mm. As a reference, the size of the stimulated area of V1—defined as the largest voxel-to-voxel distance within the region—was on average 43.7 mm (*SD* = 5.9 mm).

#### V1-patch-response-pattern analysis (Figure 2)

We tested to what extent orientation decoding relies of fine- versus coarse-scale V1 pattern components by repeating the classification analysis for V1 after subtracting out the mean activation within V1 patches (fine scale) and using only the mean activation of the patches (coarse scale). This analysis used V1 patch parcellations at different scales: 36 patch averages (corresponding to the V1 representations of the 36 stimulus patches), 12 radial-wedge averages, 4 quarter-field averages, 2 hemifield averages, and finally a single average activation for the entire representation of the stimulated region in V1.

#### Spatial high-, low-, and band-pass filtering analyses (Figure 3)

We repeated orientation decoding after spatial high-, low- and band-pass filtering. All spatial filtering operations were based on difference-of-Gaussians filtering. We smoothed the V1 activity patterns with three-dimensional Gaussian smoothing kernels of a full width at half maximum (FWHM) ranging from 1 to 40 mm in steps of 1 mm. We made sure that only information from within the stimulated part of V1 was used by treating voxels outside this ROI as missing values. The *t*-value patterns were smoothed by replacing each voxel's value by a weighted average of the surrounding voxels, where the weights are determined by a 3D Gaussian and voxels outside the ROI have weight zero. These smoothed V1 activity patterns are the low-pass filtered maps. High-pass filtered maps were computed by subtracting smoothed V1 patterns from the original unsmoothed V1 patterns—e.g., the 5-mm high-pass filtered pattern is computed by subtracting the 5-mm smoothed pattern from the unsmoothed pattern. We also created band-pass filtered patterns, with a bandwidth of 1 mm, for each spatial period by subtracting (*n* + 1)-mm smoothed patterns from n-mm smoothed patterns—e.g., the 5-mm band-passed pattern is computed by subtracting the 5-mm smoothed pattern from the 4-mm smoothed pattern. The motivation for decoding after spatial filtering is to reveal the information present in each spatial frequency band. Note that Gaussian smoothing is an invertible linear operation and therefore does not remove any information (Kamitani and Sawahata, 2010; Kriegeskorte et al., 2010). A Fisher linear discriminant will capture the effect of smoothing in its estimate of the covariance and undo this effect as it whitens the data. When using the sample covariance as the covariance estimate, a Fisher linear discriminant will therefore show identical decoding performance before after smoothing (except for deviations due to lack of numerical precision). However, linear SVMs are sensitive to information-preserving linear transformations (including non-uniform scaling) of the input variables. Our decoding results therefore are affected by smoothing and, more generally, reflect the amplitude of pattern components in different spatial frequency bands after spatial filtering.

#### Cosine models of radial and vertical preference tuning (Figure 4)

In order to analyse coarse-scale pattern effects, we computed the average response of each V1 patch representing one of the 36 log-polar stimulus tiles to each stimulus exemplar (in % signal change relative to fixation baseline). For each stimulus type and participant, we then fitted a cosine tuning model in order to estimate radial and vertical preference. The patch-response model contained a confound-mean predictor for the overall response across orientations and a cosine predictor with the peak at the hypothetically optimal stimulus orientation (radial for gratings and patch-swapped gratings, vertical for spirals and patch-swapped spirals) and the trough at the opposite orientation. We estimated radial preference for gratings (and patch-swapped gratings), but not for spirals (and patch-swapped spirals), because the latter are radially balanced. Similarly, we estimated vertical preference for spirals (and patch-swapped spirals), but not for gratings (and patch-swapped gratings), because the latter are cardinally balanced.

**Figure 4:**
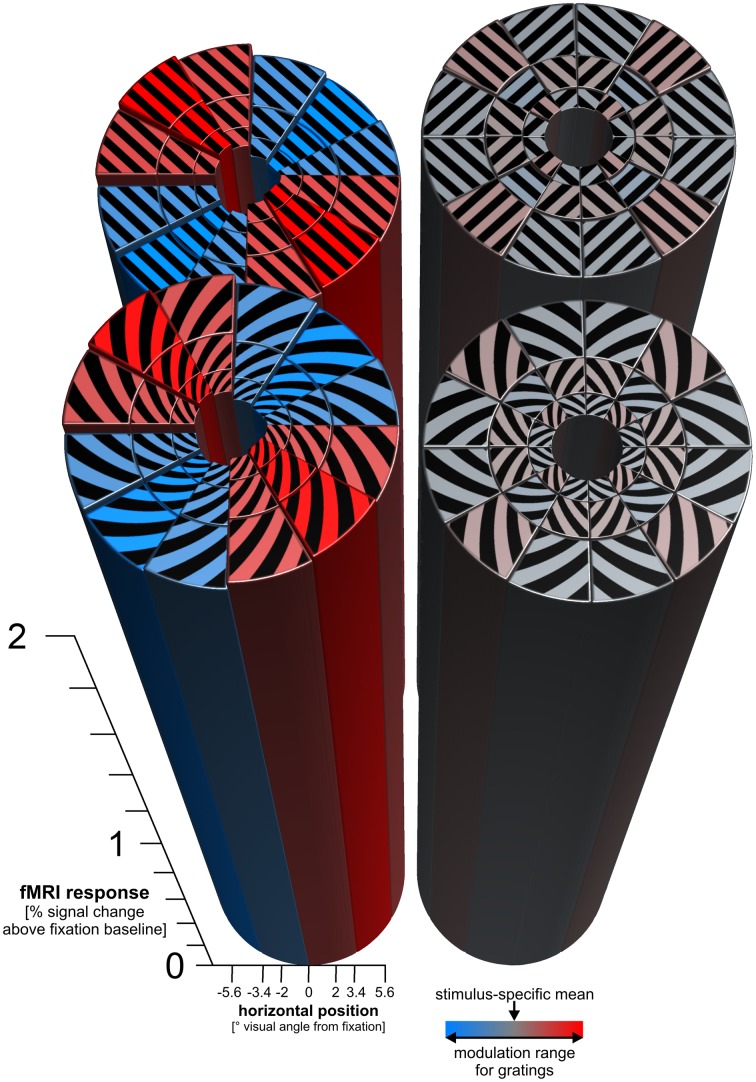
**Radial and vertical preference maps.** The figure shows the response predictions of cosine-tuning models of radial and cardinal preference. For each log-polar patch of a stimulus, a bar shows the response of the V1 region representing that patch. Each patch responds strongly to each stimulus (overall height of the bars > 2% signal change). On top of the strong overall response, there is a subtle modulation consistent with a global radial preference for gratings (± 0.025% signal change). There is a similar subtle modulation consistent with a vertical preference map for spirals (± 0.021% signal change). For patch-swapped stimuli, these modulations were much smaller (± 0.0028% signal change for patch-swapped gratings, ± 0.0018% signal change for patch-swapped spirals). These effect sizes are amplitude parameter estimates of cosine-tuning models (see Methods), averaged across all 18 participants. For inference on these effects, see Figure 5.

#### Tests for radial and vertical preference maps (Figure 5)

In order to infer whether global preference maps were present, we used the radial and vertical preference hypotheses to predict the rank order of patch responses across all patches for both exemplars (opposite orientations) of each stimulus type. Predicting the rank order requires no assumptions about the shape and width of the preference tuning. For each stimulus type, we measure the accuracy of the prediction by the rank correlation (Spearman *r*) across all patches of both exemplars in each subject. We test the null hypothesis that the preference-map prediction accuracies (one accuracy estimate per subject) are symmetrically distributed about 0 by a two-sided Wilcoxon signed-rank test. The tests are two-sided, because negative effects would indicate tangential and horizontal preference maps, respectively, and we did not intend to exclude these possibilities *a priori*. To compare the strength of the preference-map prediction accuracies between stimulus types, we computed the accuracy difference for each subject and performed the same two-sided test on the accuracy differences.

**Figure 5:**
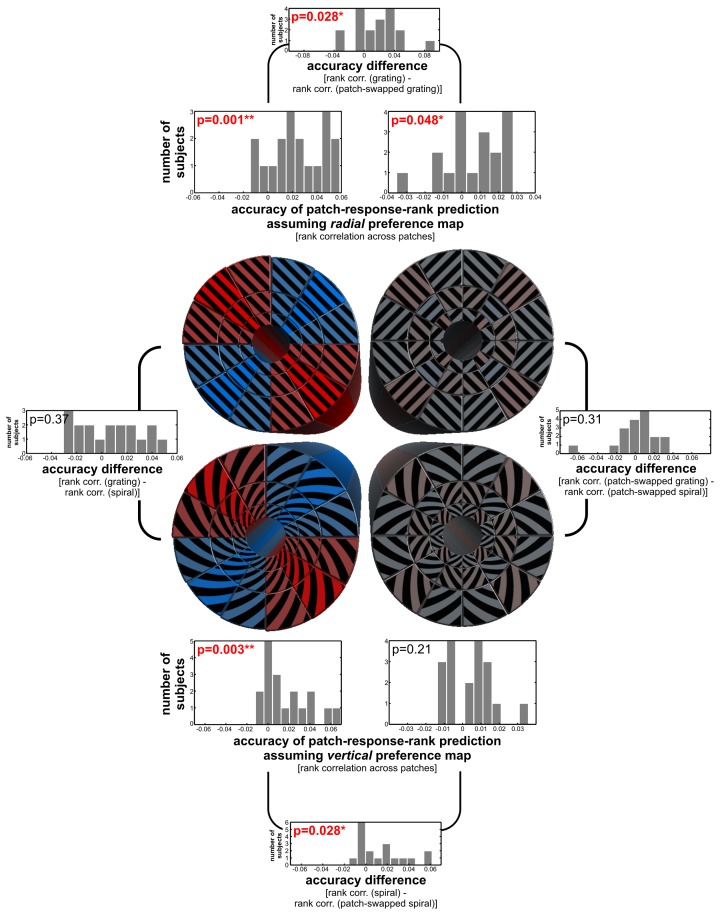
**Statistical inference for radial and vertical preference maps and preference differences.** Inference is based on the accuracy with which the radial/tangential or vertical/horizontal preference model predicts the rank order of response amplitudes across patches (see Methods). The histograms show the distribution across subjects of these accuracies (Spearman *r*). We performed a two-sided Wilcoxon signed-rank test on each accuracy distribution. The *p*-value for each effect is in the top left corner of the corresponding histogram and in bold red if it indicates a significant effect. The outer plots show differences between stimulus types in the strength of radial and vertical preference effects, and their *p*-values from the same two-sided signed-rank test.

#### Effect of subject head motion on orientation decodability (Figure 6)

We investigated the effect of head motion on orientation decodability by calculating a head-motion index for each subject and correlating this index with orientation-decoding accuracy across subjects. We used the decoding accuracy for V1 averaged across all stimulus types. The head-motion index was computed by averaging translation (in mm) and rotation (in deg) estimates from the head-motion correction algorithm. These estimates were averaged across across time (fMRI volume) and across all three parameters (dimensions of translation and axes of rotation, respectively). This index is based on the fMRI data for the eight runs during which all four stimulus types were presented. To test for differential effects of head motion on fine versus coarse spatial frequency patterns, we repeated this analysis after band-pass filtering at spatial periods ranging from 1 to 40 mm.

**Figure 6:**
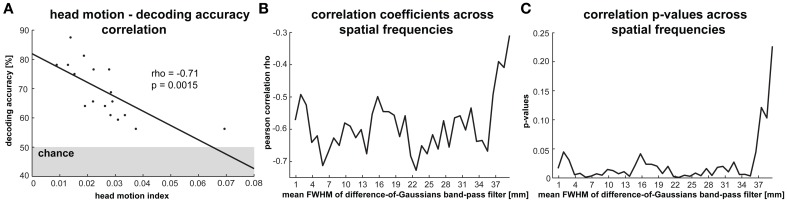
**Impact of head motion on orientation decodability. (A)** The correlation between a head motion index—reflecting the average translation and rotation changes per volume/2 s—and average orientation classification performance (averaged across all stimulus types) across participants. There are 17 data points because we excluded one participant whose average head motion was more than three standard deviations greater than the group average. **(B)** Pearson correlation coefficients and **(C)** *p*-values for the correlation between head motion and decoding performance based on activity patterns that were band-pass filtered at spatial frequencies ranging from 1 to 40 mm.

## Results

### Globally coherent stimuli are highly decodable, but spirals with lower accuracy than gratings

Figure 1B shows the results for orientation decoding based on V1, V2, and V3 response patterns. The orientation of uniform visual gratings could be decoded from V1, V2, and V3 with high accuracy (77% for V1, *p* < 0.0005, Wilcoxon signed-rank test on the set of single-subject accuracy estimates), replicating the classical finding (Haynes and Rees, 2005; Kamitani and Tong, 2005). Radially balanced spirals could be robustly decoded as well (69% for V1, *p* < 0.0005). This generalizes a similar finding for radially balanced spiral Glass patterns (Mannion et al., 2009) and suggests that a radial-preference map does not fully account for orientation decodability. However, decoding was significantly less accurate for spiral stimuli than for grating stimuli (*p* < 0.05, Wilcoxon signed-rank test on the set of single-subject accuracy differences), which is consistent with a contribution to decoding from a radial-preference map (Sasaki et al., 2006; Freeman et al., 2011).

### Patch-swapped stimuli are robustly decodable, but at lower accuracy than globally coherent stimuli

The opposite-orientation exemplars of patch-swapped gratings and patch-swapped spirals could be robustly decoded from V1 (64%, *p* < 0.005 for patch-swapped gratings; 62%, *p* < 0.01 for patch-swapped spirals). The patch-swapped stimuli were also significantly decodable from V2 and V3. Global-form differences, thus, are not necessary for two orientation stimuli to be discriminable from early visual fMRI patterns. However, decoding accuracy estimates were lower for patch-swapped than for globally coherent stimuli in V1, V2, and V3. This effect was significant for gratings in V1 (*p* < 0.05). This suggests that global-form differences contribute to orientation decodability. The profile of decoding accuracies was not significantly different between early visual areas V1, V2, and V3 (*p* > 0.25, repeated-measures ANOVA interaction between visual area and stimulus-dependent effects).

### Fine-scale pattern components enable high-accuracy orientation decoding for all stimulus types

#### Decoding after subtracting V1-patch averages

In order to determine the spatial grain of the fMRI pattern components that contribute to orientation decoding, we divided V1 into equally sized patches at different scales (Figure 2A). To test if within-patch fine-scale V1 patterns are sufficient for decoding orientation, we removed the coarse-scale pattern from each fMRI pattern before decoding. For each fMRI pattern, we computed the patch average activations (capturing the coarse-scale pattern component) and subtracted that component from the V1 pattern (Figure 2A), thus shifting the fine-scale pattern in each patch to an average activation of 0. This had no significant effect on decoding accuracy even at the finest scale (36 segments). Accuracy remained significantly above chance (*p* < 0.01) for all stimulus types and scales. This suggests that coarse-scale components might not be necessary for orientation decoding.

#### Decoding after spatial high-pass filtering

To further explore the spatial grain necessary for orientation decoding, we performed a continuous spatial high-pass filtering analysis (Figure 3A). Gratings are highly decodable (decoding accuracy = 60.0%, *p* < 0.0003) even after 1-mm-FWHM high-pass filtering. The other stimulus types also all rapidly rise to decodability around 2-mm-FWHM of the high-pass filter. Pattern components at a fine scale, matching the voxel size (2 mm), thus, appear to suffice for decoding orientation stimuli. This finding provides further evidence that orientation decoding does not require coarse-scale pattern components.

#### Decoding after spatial band-pass filtering

In order to determine the degree to which each spatial scale contributes to the decoding of each stimulus type, we performed decoding after spatial band-pass filtering (Figure 3C). Consistent with the high-pass analysis, we found significant decodability for all stimulus types at very fine spatial scales approximately matching the voxel size (2 mm). All stimulus types were optimally decodable at a spatial scale of about 5 mm. Interestingly, the decoding accuracies achieved in this band (>80% for gratings, around 70 % for the other stimulus types) matched or exceeded those in all other analyses, including those of high-passed, low-passed, and unfiltered data. This suggests that the signal-to-noise ratio of the orientation information might be best in this band. Patch-swapped stimuli became progressively less decodable at coarser scales, whereas globally coherent stimuli remained robustly decodable at coarse scales (25–35 mm FWHM). This is consistent with global radial (for gratings) and cardinal (for spirals) preference maps, which predict that V1 should show a checkerboard-like alternation between the response patterns expected for the globally coherent parents of the patch-swapped stimuli. However, the actual V1 activation patterns driving this effect suggest that the global preference maps are also weaker or absent for patch-swapped stimuli (Figures 4, 5, results described below).

The spatial band-pass analysis additionally indicated that globally coherent gratings were significantly more accurately decodable than all other stimulus types at the finest spatial scales from 1 to 6 mm (*p* < 0.05). This suggests that the decoding advantage for gratings versus spirals described above results from a greater amount of information in fine-scale patterns for gratings. Gratings were more decodable than patch-swapped stimuli across all frequency bands. Interestingly, the spirals' decodability grouped with patch-swapped stimuli in the high spatial-frequency band and with the gratings in the low spatial-frequency band (see also Figures 2A,B and 3A,B). In the low band, spirals might be similarly decodable as gratings, because each globally coherent stimulus type benefits from a global preference map (cardinal for spirals, radial for gratings). That spirals (like patch-swapped stimuli) are less decodable than gratings in the high band is harder to explain. We will revisit this point in the Discussion.

### Coarse-scale pattern components enable orientation decoding, but at lower accuracies, especially for patch-swapped stimuli

#### Decoding based on V1-patch averages

To measure coarse-scale information, we decoded orientation using the patch-average activations only, thus removing the fine-scale component from each fMRI pattern (Figure 2B). This analysis started with 36 patch averages (corresponding to the V1 representations of the 36 stimulus patches) and progressed to 12 radial-wedge averages, 4 quarter-field averages, 2 hemifield averages, and finally a single average activation for the entire representation of the stimulated region in V1. Consistent with the band-pass analysis, patch-swapped stimuli became progressively less decodable at coarser scales and were no longer significantly decodable at the quarter-field scale (*p* = 0.56, *p* = 0.78, for patch-swapped gratings and spirals, respectively). Decoding accuracy also declined for globally coherent stimuli, but remained robustly significant at the quarter-field scale (*p* < 0.005, *p* < 0.05, for gratings and spirals, respectively). At this scale, decoding accuracy was significantly greater for globally coherent than for patch-swapped stimuli (*p* < 0.05). This is consistent with a contribution from global preference maps to the decoding of globally coherent stimuli as well as patch-swapped stimuli (where the global-map hypothesis predicts contrast alternation from patch to patch). Accuracy was not significant for any stimulus type when only each hemifield's average or the overall average of the V1 representation of the stimuli was used.

#### Decoding after spatial low-pass filtering

The spatial low-pass filtering analysis (Figure 3B) similarly shows a decline of decodability of patch-swapped gratings and patch-swapped spirals at coarse scales. Patch-swapped stimuli were no longer significantly decodable when V1 response patterns were smoothed with a Gaussian kernel of 12 mm FWHM or wider. Globally coherent stimuli, by contrast, remained decodable after smoothing the patterns with Gaussians of up to 24 mm FWHM. As a reference, the size of the stimulated area of V1—defined as the largest voxel-to-voxel distance within the region—was on average 43.7 mm (*SD* = 5.9 mm).

### Globally coherent stimuli reveal subtle but significant radial and vertical biases, but these appear weaker or absent for patch-swapped stimuli

The decoding analyses just described indicated the presence of coarse-scale information, but did not reveal whether the actual coarse-scale patterns are consistent with biases in favor of radial or cardinal orientations. We fitted a cosine tuning model (see Methods) to the responses of the 36 V1 patches (i.e., the representations of the 36 log-polar stimulus patches in V1) in order to estimate the global radial and vertical preferences for each stimulus type. The fitted models' predicted global response patterns are shown in Figure 4, for parameters averaged across our 18 subjects. Radial and vertical preference maps were evident for globally coherent gratings and spirals respectively which is in line with previous studies (Freeman et al., 2011; Merriam et al., 2012). These preference maps, however, were very subtle (approximately ± 0.02 % signal change) compared to the overall response above fixation baseline of each V1 patch to any orientation (>2% signal change). For patch-swapped stimuli, radial and vertical preference modulations were about an order of magnitude smaller than for globally coherent stimuli.

We performed statistical inference to test for radial/tangential and vertical/horizontal preference maps and to compare the strength of these global preference maps between stimulus types (Figure 5). We used the radial and vertical preference hypotheses to predict the rank order of responses across all patches (see Methods). Negative effects would have indicated tangential or horizontal preferences, respectively, but were not observed at the level of group averages. Response modulation consistent with radial and vertical biases was significant for globally coherent gratings and spirals (*p* = 0.001, *p* = 0.003, respectively, two-sided Wilcoxon signed-rank test across single-subject effects). For patch-swapped stimuli, the radial and vertical preference effects were significantly weaker (*p* < 0.03 for both comparisons). The radial preference effect was still significant for patch-swapped gratings (*p* < 0.05), but the vertical preference effect was not significant for patch-swapped spirals. We found no significant preference effect differences between gratings and spirals, or between their patch-swapped variants.

These results suggest that the strength of radial and vertical preferences depends to some degree on the spatial coherence of the orientation stimuli. This would be consistent with extra-receptive field effects related to preattentive grouping, attention spreading, or global-form perception. Such effects could arise through recurrent processing via long-range intrinsic connections or via feedback from higher stages of representation. However, we were concerned that inaccuracies of the definition of the V1 patches might have artifactually reduced the apparent strength of the global-preference maps when analysing responses to patch-swapped stimuli. If a V1 patch were incorrectly defined, so as to straddle the boundary between two patches, the signals from the two sampled patches would mix and opposite responses would cancel out to some degree, lowering the contrast between the exemplars. This effect would be less of a problem for globally coherent stimuli, where adjacent patches are stimulated with similar orientations and we thus expect a smooth variation of the response across patches. In order to reduce the influence of V1-patch-definition inaccuracies and test for global-preference maps with maximum sensitivity, we performed a control analysis, excluding low-contrast patches. For each stimulus type, we initially considered all patch responses, as before. For each patch in each subject, we computed the patch-response contrast between the two exemplars (positive for exemplar 2 > exemplar 1, negative for exemplar 1 > exemplar 2). We then correlated the actual patch contrasts with the predicted patch contrasts based on the preference map hypothesis using Spearman's *r*, as before. We repeated this procedure excluding the patches with the lowest contrast between the two exemplars. For globally coherent stimuli, preference maps always provided significant patch-contrast predictions when the lowest-contrast 0–85% of the patches was excluded. For patch-swapped gratings and spirals, however, there was never any significant patch-contrast prediction—even without correction for multiple testing. This suggests that the weak evidence of preference maps for patch-swapped stimuli is not an artifact of inaccurate V1-patch definitions.

### Head motion appears to strongly reduce orientation decodability

Head motion is expected to reduce the reliability of response-pattern estimates. Even after rigid-body head-motion correction, residual head-motion-related artifacts remain. It has been suggested that these might selectively reduce pattern information in the high spatial frequency band (Kriegeskorte et al., 2010; Swisher et al., 2010). To assess the effect of head motion, we determined the correlation between a head-motion index computed for each subject (an average of translations and rotations between successive fMRI volumes, see Methods) and V1-based orientation decoding performance (averaged across the four stimulus types). We excluded one participant whose average head motion was more than three standard deviations greater than the group average. Our data show a significant negative relationship between head motion and orientation decodability (*r* = −0.7, *p* = 0.0015, Figure 6A). Orientation decoding accuracy ranged from 76 to 88% for the five participants with the lowest head motion indices while accuracies ranged from 56 to 61% for the five participants with the highest head motion indices. This suggests that head motion strongly reduces pattern decodability. Alternatively, or in addition, subjects who moved more may also have been less attentive to the task and/or less reliable in fixating the central dot throughout the experiment.

In order to test if head motion selectively affected orientation information in the high spatial-frequency band, we repeated this analysis for V1 activation patterns that were spatially band-pass filtered. As before, we used difference-of-Gaussians filters ranging from 1 to 40 mm FWHM (with 1 mm difference in FWHM between the two Gaussians). We plotted the correlation coefficients between the head-motion index and decoding accuracy and the corresponding *p*-values as functions of spatial frequency (Figures 6B,C). The head-motion index was significantly negatively correlated with decoding accuracy in all but the very lowest spatial frequency bands (> 36 mm FWHM), in which none of the stimulus types could be reliably decoded. Our results, thus, do not support the claim that residual head-motion artifacts (after head-motion correction) affect pattern information selectively in the high spatial-frequency band.

### The average V1 response declines with eccentricity, but appears unaffected by stimulus type and polar angle

#### Univariate effect of stimulus type

**Figure 7:**
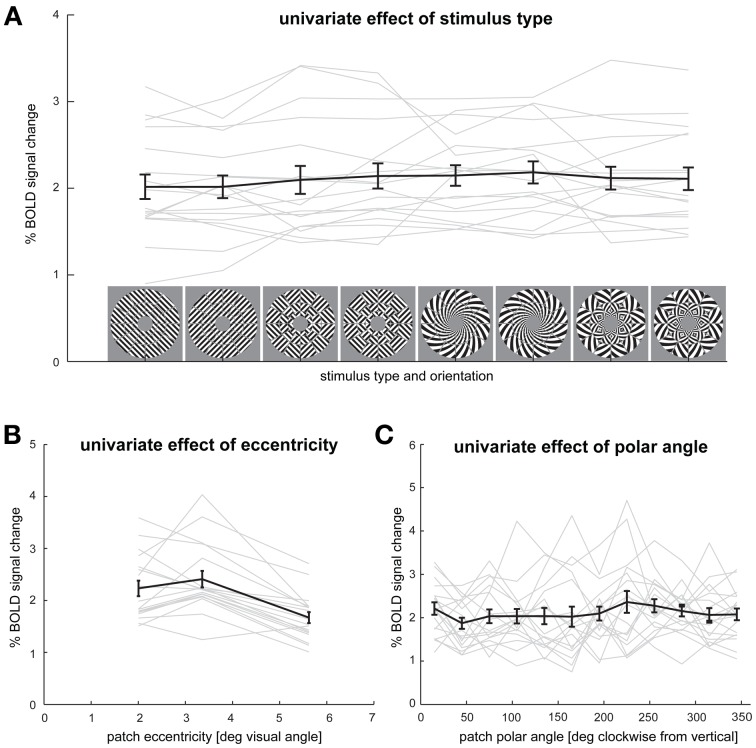
**Effects of stimulus type, eccentricity, and polar angle on average V1 responses. (A)** Spatial-mean V1 activation for each stimulus type and orientation. **(B)** V1 response for each of the three patch eccentricities. **(C)** V1 responses for each of the 12 polar angles. Responses are averaged across the remaining variables (stimulus type, V1 patches, participants). Error bars indicate the SEM computed across participants. Gray lines depict each individual participant's responses.

It is possible that differences in orientation decodability resulted from differences in the signal-to-noise ratio of V1 responses across stimulus types (Tong et al., 2012). To test for this possibility, we compared the average % signal change in V1 across all stimulus types. Mean percent signal change was 2.02 (*SEM* = 0.13) for grating stimuli, 2.12 (*SEM* = 0.15) for patch-swapped grating stimuli, 2.16 (*SEM* = 0.12) for spiral stimuli and 2.11 (*SEM* = 0.13) for patch-swapped spiral stimuli (Figure 7A). We observed no significant differences in mean percent signal change in V1 between stimulus types.

#### Univariate effects of eccentricity and polar angle

We also tested if average V1 patch responses differed as a function of patch eccentricity and patch polar angle after averaging patch responses across all stimulus types (Figures 7B,C). The only effect we found in these analyses was that the average response for patches in the inner and intermediate (2.0° and 3.4°, respectively) rings were greater than those for patches in the peripheral (5.6°) ring (*p* < 0.01).

### Orientation decoding is less robust for peripheral than central patches for globally coherent gratings

**Figure 8:**
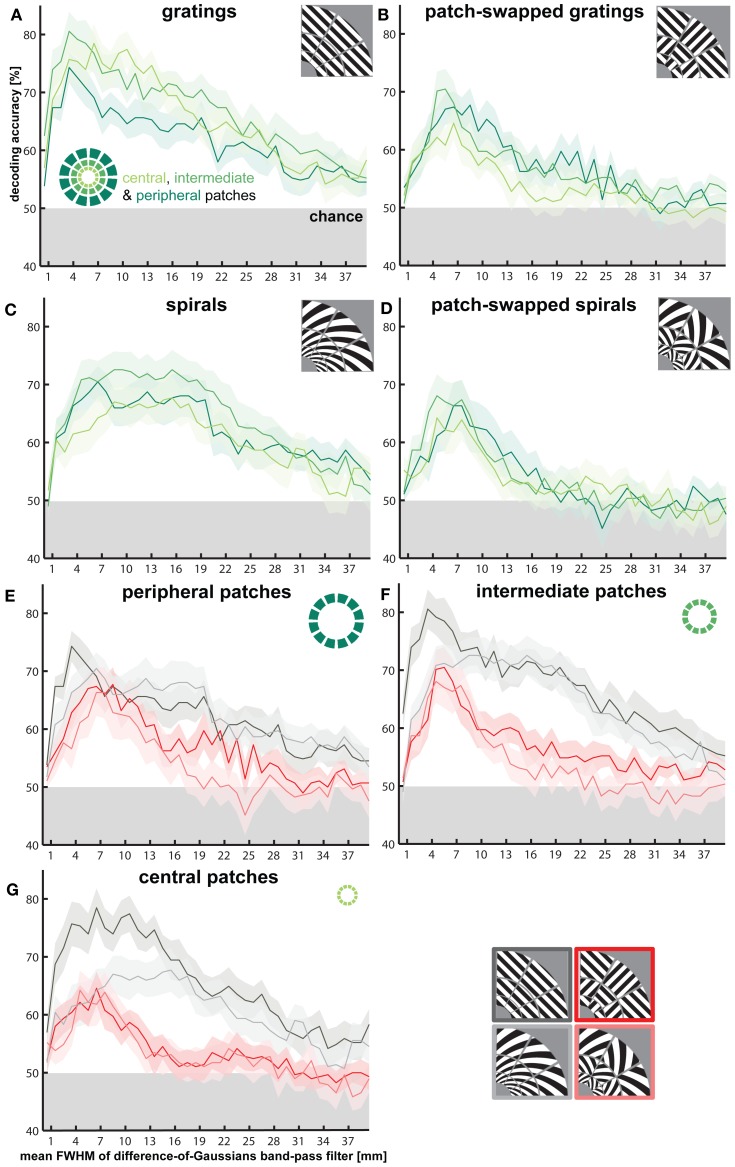
**Effects of V1 patch eccentricity on orientation decodability.** Same band-pass filtering approach as for Figure 3C, but plotted separately for patterns in the central, intermediate, and peripheral ring of V1 patches. **(A–D)** highlight differences in orientation decodability between rings, depicted for each stimulus type separately. **(E–G)** highlight differences in orientation decodability between stimulus types, depicted for each ring separately.

To test whether lower average responses in peripheral V1 patches is associated with reduced orientation decodability, we assessed decodability for all stimulus types using central (2° eccentricity), intermediate (3.4°) and peripheral (5.6°) V1 patches (Figure 8). For each ring of patches, we analyzed decodability as a function of spatial frequency. The only significant effect was a reduction of accuracy when decoding gratings from the peripheral patches for intermediate spatial frequencies (Figure 8A, note overlapping error margins in Figures 8A–D). Orientation decodability was lower for peripheral as compared to intermediate and central patches for spatial bands corresponding to Gaussian widths from 6.5 to 15.5 mm (*p* < 0.05, for all band-pass accuracy differences, except the accuracy difference at 12.5-mm, Figure 8A). We did not observe any other significant effects of eccentricity on decoding accuracy.

### Patch-swapped stimulus pairs are perceived as more similar than their globally coherent parent stimuli

We asked 13 subjects (separate group from those in the fMRI experiment) to judge the visual dissimilarity of our stimuli (see Methods). Subjects judged patch-swapped stimulus pairs to be more visually similar than their globally coherent parents (*p* = 0.0043 for gratings, *p* = 0.038 for spirals, signed-rank test). We did not observe a significant difference in perceptual dissimilarity between (a) the pair of gratings and the pair of spirals (*p* = 0.22) or (b) the pair of patch-swapped gratings and the pair of patch-swapped spirals (*p* = 0.21). These results support the idea that patch-swapping, despite preserving the local orientation disparity throughout the spatial extent of the stimulus, reduces perceptual dissimilarity.

## Discussion

### Coherent global form may contribute to apparent global preference maps

Orientation stimuli differing in global form (spirals and gratings) were more distinct in V1 fMRI patterns than stimuli with similar global form (patch-swapped variants). This suggests that global-form differences contribute to fMRI orientation decoding. Patch-swapped stimulus pairs were not only less decodable, but also had weak or absent global radial and vertical biases. While we found some evidence for a radial preference map for patch-swapped gratings, this effect was about an order of magnitude smaller than for globally coherent gratings. (And even for globally coherent gratings, the global preference map constituted a very subtle modulation of the overall V1 response, as shown in Figure 4.) This suggests that global radial and vertical preference maps in V1 are subtle and might depend to some extent on the degree of global coherence of the stimulus.

Coherent global form could contribute to global preference maps in a number of ways. First, attention might automatically spread along the edges of the grating emanating radially from fixation (Wannig et al., 2011). This would produce higher V1 responses for radial than for tangential parts of a grating. Second, representations of global form in higher visual areas (Pasupathy and Connor, 2002; Kourtzi and Huberle, 2005; Ostwald et al., 2008) might give rise to coarse-scale reflections of global form in V1 via feedback connections. Third, interactions between center and surround of V1 receptive fields (e.g., Sengpiel et al., 1997) might produce global-scale response variation. In particular, the orientation of the edge of the ring-shaped mask of the grating might interact with the orientation of the grating itself. Where the grating orientation is radial, the edge of the ring is orthogonal to the grating; where the grating orientation is tangential, the edge of the ring is parallel to the grating orientation. This would suggest greater surround inhibition for the tangential part of the grating, which could produce an apparent radial bias (Freeman et al., 2011). A smooth global-scale variation is not expected to arise from center-surround interactions for more complex stimuli, including natural images and patch-swapped gratings and spirals, where there are multiple orientations surrounding each location.

As a caveat to the interpretation of our findings, imprecise fixation might have differentially affected the representation of globally coherent and patch-swapped stimuli. Small eye movements might have effectively blurred the V1-patch checkerboard entailing greater reduction of decoding contrast for patch-swapped than for globally coherent stimuli. This might also have reduced the apparent strength of radial and vertical preference maps for patch-swapped stimuli. Although such an effect of eye movement cannot be completely ruled out, note that our task required continual fixation to discern tiny foveal stimuli. Successful performance suggests that lapses of fixation were minimal. As a second caveat, imprecision in V1-patch definitions might have led to reduced patch-response contrast for patch-swapped stimuli, where the preference predicted by the global map inverts for adjacent patches. However, a control analysis suggested that the drop in global-preference effect strength for patch-swapped stimuli was not due to imprecise patch definitions. When we excluded the lowest-contrast 5–95% of the patches (i.e., those most likely to be imprecisely defined), there was never a significant correlation (Spearman *r*) between the measured patch contrast for two opposite-orientation patch-swapped stimuli and the patch contrast predicted by the global-preference-map hypothesis (radial for patch-swapped gratings, vertical for patch-swapped spirals). In other words, even the highest-contrast patches showed no evidence for a global-preference map for either patch-swapped gratings or patch-swapped spirals.

Beyond the specific results of the present study, the spatial and temporal coherence of orientation stimuli is likely to affect their representation in early visual cortex. Previous studies have used gratings, which are spatially coherent (single orientation) and spatially continuous (edges unbroken). In some studies, the grating orientations were also temporally continuous (a rotating grating; e.g., Freeman et al., 2011). A rotating grating elicits the percept of a rigid moving object and will deeply engage multiple levels of the visual hierarchy. The entirely predictable spatial and temporal structure of the display is expected to trigger recurrent and predictive representations, in which top-down and lateral recurrent signals interact richly with the visual input fed forward from the retina. Effects observed with such stimuli do not support simple interpretations in terms of feedforward processing (Gilbert et al., 1996; Stettler et al., 2002). Spatial coherence (constant or smoothly changing orientation) and continuity (unbroken edges) as well as temporal coherence and continuity (stimuli presented in a smooth continuous rotation, rather than in random order) are expected to affect perceptual grouping and automatic attention spreading as well as the higher-level representation of the stimulus and recurrent top-down effects on the early visual representation.

In conclusion, then, it is plausible that feedforward, classical-receptive-field responses in V1 exhibit radial- and vertical-preference maps, reflecting the natural frequency of different orientations in the visual patterns impinging on the retina (Furmanski and Engel, 2000). However, previous studies suggesting global preference maps (Sasaki et al., 2006; Freeman et al., 2011) used globally coherent orientation stimuli that are not well-suited for ruling out alternative explanations based on extra-classical receptive fields, attention spreading, and top-down influences from global-form representations. Our study suggests that coherent global form contributes to global preference maps to some extent, which is in line with a recent finding by Mannion et al. (2010b). Future studies using complex non-coherent stimuli, including natural images, and random stimulus sequences will be required to characterize the nature of global orientation-preference maps in early visual cortex.

### Fine-grained components of fMRI patterns are sufficient for orientation decoding

If fMRI orientation decoding relied exclusively on global preference maps (Freeman et al., 2011), we would expect greatest decoding accuracy for spatially low-passed fMRI patterns, because fine-grained voxel-to-voxel variations would contribute more noise than signal. However, decoding accuracy degrades with spatial low-pass filtering and is preserved for high-pass filtering up to very fine scales. This indicates that fine-grained voxel-to-voxel signal variations do carry much information about visual orientation.

The V1 orientation-preference map is a complex irregular pattern of orientation columns which combines variations at different spatial scales, from sharp discontinuities of preference, through rapid continuous changes of preference around pinwheels, and possibly on to subtle global-scale biases in the relative number of neurons preferring each orientation (Sasaki et al., 2006; Mannion et al., 2010a; Freeman et al., 2011). In the spatial frequency domain, the V1 orientation preference map is therefore likely to have significant energy in a wide range of spatial frequencies. At high field strength, high-resolution fMRI can directly visualize the columnar pattern (Yacoub et al., 2008). At 3T and lower spatial resolutions, fMRI may reflect preference variations in slightly lower spatial frequencies.

The present results do not speak to question of fMRI hyperacuity (Gardner, 2010; Kamitani and Sawahata, 2010; Kriegeskorte et al., 2010; Op de Beeck, 2010a,b; Shmuel et al., 2010). Hyperacuity would mean that fMRI patterns reflect neuronal patterns at subvoxel scales, i.e., spatial frequencies above the Nyquist limit imposed by voxel size. This possibility is suggested by the fact that an fMRI voxel samples the neuronal activity patterns through a complex spatiotemporal transform, which is unlikely to be well described by a local-averaging model (Kriegeskorte et al., 2010). A complex spatiotemporal filter could have some sensitivity to spatial frequencies above the Nyquist limit. Addressing how strongly fMRI patterns (at a given voxel width) actually reflect neuronal pattern energy at scales finer than the voxels would require experiments that enable us relate neuronal patterns at these sub-voxel scales to fMRI patterns without confounding effects at larger scales. Until this difficult experimental challenge is met, the question of fMRI hyperacuity remains open.

### Spatial filtering can reveal how components at different scales contribute to fMRI pattern contrast, but the interpretation requires some caution

Several studies have used decoding after spatial filtering of fMRI patterns (Op de Beeck, 2010a,b; Shmuel et al., 2010; Swisher et al., 2010). Our results further illustrate the usefulness of such analyses for understanding the spatial structure of the fMRI pattern information and how it varies across different types of stimulus. However, several complications have to be considered in interpreting decoding results obtained after spatial filtering of fMRI patterns.

First, Gaussian smoothing reduces the amplitude of high-spatial-frequency components, but it does not reduce the information content of the patterns (Kamitani and Sawahata, 2010; Kriegeskorte et al., 2010). The reason for this becomes apparent when considering the frequency-domain representation of the pattern. Convolution with a Gaussian in the space domain is equivalent to multiplication with a Gaussian (whose width is proportional to the reciprocal of the width of the original Gaussian) in the frequency domain. While this scales down high-frequency components, it does not scale any frequency range down all the way to zero. As a consequence, high-spatial frequencies can simply be scaled back up, inverting the filter. Equivalently, the convolution can be performed by a matrix multiplication. This linear recoding entails greater correlations among voxels in the same neighborhood, but the number of voxels and the intrinsic dimensionality of the data is preserved. Multiplication with the inverse matrix will exactly recover the original data. A Fisher linear discriminant using the sample covariance will exactly invert the smoothing transform and yield identical results (if numerical precision is sufficient) before and after Gaussian smoothing of the patterns (Kriegeskorte et al., 2010). However, if we assume a diagonal covariance with the Fisher discriminant or use a linear SVM for decoding (as we have done here), results are not equivalent and do predominantly reflect the pattern contrast in the spatial-frequency bands that most prominently pass the filter. Note that downsampling analyses, like that of Figure 2, where we replaced the pattern by a set of patch averages, also do reduce the information content (i.e., the original pattern cannot be recovered from the patch averages). Such analyses therefore provide a useful alternative perspective.

Second, Freeman et al. (2011) found that decoding accuracy was significant after highpass filtering for both the orientation of a grating and the polar angle of a contrast-rich wedge. Because the polar angle map is presumably smooth, they argued that highpass filtering results are difficult to interpret. However, the polar angle map is expected to contain a wide range of spatial frequencies. In particular, polar angle changes more rapidly in the central part of the representation. Moreover, the wedge stimulus they used in fact has sharp edges, so the spatial response pattern it elicits in a smooth retinotopic map is expected to have sharp edges as well. The continuous motion of the wedge does suggest that the response should be smoothed according to the shape of the hemodynamic response, with each fMRI volume reflecting a range of positions to different degrees. However, all these complications make it difficult to draw firm conclusions on utility of spatial filtering from Freeman et al. (2011). Importantly, our results show significant differences between stimulus types in terms of their decodability from different spatial-frequency bands. In sum, spatial filtering analyses are an important tool for understanding the spatial structure of fMRI pattern effects, but their interpretation requires some caution.

## Conclusions

**Figure 9:**
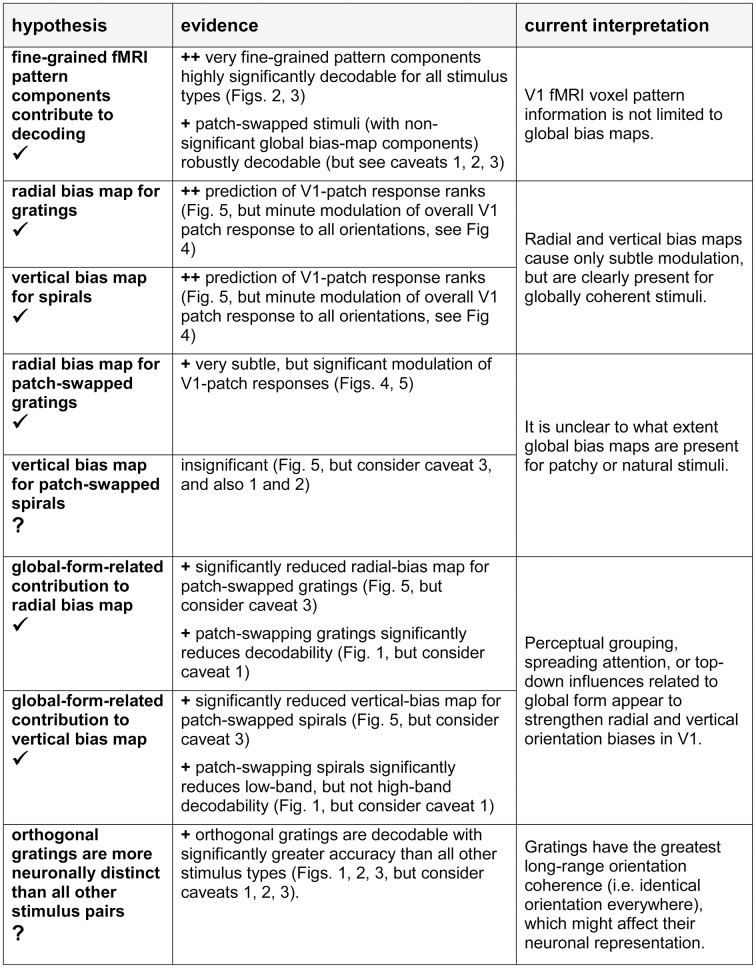
**Hypotheses, evidence from this study, and our current interpretation**. In the hypothesis column, a checkmark indicates the presence of significant evidence in favor of the hypothesis; a question mark indicates that the evidence is weak or absent. In the evidence column, ^++^ indicates strong evidence, ^+^ indicates evidence to be considered in the light of potential caveats. Caveats: (1) Imprecise fixation may have blurred the V1-patch checkerboard entailing greater reduction of decoding contrast for patch-swapped than for globally coherent stimuli. (2) Imprecise fixation may also have added noise to the local orientation signal for all stimuli, except gratings. However, our task required continual fixation to discern tiny foveal cues. Successful performance suggests that lapses of fixation were minimal. (3) Imprecision in V1 patch definitions might have led to reduced patch contrast for patch-swapped stimuli, where the preference predicted by the global map inverts for adjacent patches. However, a control analysis (see Results, Discussion) did not support this account.

Figure 9 presents an overview of the main findings of this study and our current interpretation. The fine-grained component of fMRI response patterns clearly reflects the representation of visual orientations in V1, suggesting that fine-grained fMRI patterns, down to the scale of individual voxels, reflect neuronal pattern differences. This result holds not only for spatially coherent orientation stimuli (like gratings), but also for more complex stimuli combining multiple orientations, and in the absence of strong global-form differences between the decoded stimuli. These findings support the idea that fMRI orientation decoding reflects local orientation-specific responses rather than global-percept related top-down signals. The spatial and temporal coherence of the stimuli is expected to affect perceptual grouping and automatic attention spreading as well as the higher-level representation of the stimulus and recurrent top-down effects on the early visual representation. Globally coherent stimuli are therefore not ideal for studying the feedforward-based component of the representation in early visual cortex. Future studies using incoherent orientation stimuli will be needed to address the degree to which the radial and cardinal orientations are overrepresented in early visual orientation preference maps even in the absence of local recurrent and feedback effects related to perceptual grouping and global stimulus representation at higher stages of the visual system.

### Conflict of interest statement

The authors declare that the research was conducted in the absence of any commercial or financial relationships that could be construed as a potential conflict of interest.
